# Vicarious Menstruation

**Published:** 1855-12

**Authors:** J. Boring

**Affiliations:** Prof. of Obstetrics, &c., in the Atlanta Medical College


ARTICLE V.—*Vicarious Menstruation.* By J. BORING, M. D., Prof. of Obstetrics, &c., in the Atlanta Medical College.

The subject of menstruation, together with every deviation from the healthy performance of this function, must, in the very nature of things, always excite the interest of the physician who feels, as he should, the responsibility of his profession.

While there are *extreme* exceptions, it holds good, as a general rule, that without healthy menstruation, the human female is incapable of the high function of her species. It is also true, and but too little understood and appreciated, that with menstrual derangement, health of body and mind are not to be expected. So intimately is this peculiar function connected with the sex, and interwoven with its very existence, that life cannot indeed be regarded as fully established without it. Every departure from the healthy standard must therefore be regarded with deep interest.

Every physician of anything like extended practice, knows the painful extent and diversity of what are commonly called "Female Complaints," "Armenorrhæa," "Dysmenorrhæa," "Menorrhagia," "Leucorrhœa," etc., and the extreme difficulty of perfecting a cure.

"Vicarious Menstruation," the subject now under consideration, is perhaps amongst the most singular and intractable derangments of menstrual secretions. That the fact exists, will, it is presumed, be denied by no intelligent physician, but why, or how one organ, or member of the body, should assume and perform the functions of another, and those too of such widely different offices in their normal conditions, are questions of difficult solution. The uterus may, with great propriety, be held to be an organ *sui generis*, both in its structure and physiological nature; and yet, when deranged by disease, it has been known, that the eyes, the nose, breasts, fingers, toes, skin, etc., have taken on the menstruating office, and performed that peculiar function, with as regular movements as those of the womb itself. It is true, that in most instances of Vicarious Menstruation, sterility obtains, but this fact does not, in general, seem to interrupt the periodic return of the catemenial effort of nature, or the normal attendant symptoms, such as backache, headache, heaviness, etc. There is obviously a preparation of nature for the discharge of this function, but owing, as is believed, to some uterine derangement, that organ is incapacitated for the work, and by an infinitely wise provision of Providence, the system is relieved by the action of some other member or organ of the body. I have said before, and now repeat, that this phenomenon arises from uterine derangement. It is, in my judgement, important that this view should be distinctly impressed upon the mind of the physician. *It is a diseased action*, and should be so treated. It will be found in most, perhaps all cases of the kind, that the womb is the subject of chronic congestion, inflammation, or ulceration, and hence its incapacity for the performance of this peculiar function.

From the foregoing it will follow, of course, that for the correction of this singular departure from healthy action, the treatment should be directed to the uterine system. Time will not allow a further prosecution of this branch of the subject at present, and I shall therefore conclude by the statement of the facts in two cases of Vicarious Menstruation, which have recently come under my observation and treatment.

The first is, that of a married lady, Mrs. ——, twenty-two or three years of age, of rather feeble constitution, sensitive, nervous habit, poor digestion, and generally feeble health.—She menstruated first at the usual age and continued healthy in this respect for the space of three or four years, when the catemenial flow was suddenly arrested by falling into a stream of water, the discharge being present at the time. Since then she has been the subject of irregular menstruation, the secretion coming on sometimes too soon, then too late; at times insufficient in quantity, and at other periods profuse and exhausting. She has been married about two years, but without issue. When I first saw her, she was laboring under what was supposed a dangerous attack of hemorrhage from the stomach and bowels. I found her vomiting and purging what seemed to be decomposed blood, with great exhaustion, and the pulse ranging from 120 to 125 in the minute. It did not occur to my mind at first, that this was an instance of Vicarious Menstruation, but the physician who had been in charge of the case, having blistered over the region of the stomach, and administered the usual remedies, I determined to wait and extend my observations before instituting treatment. In the mean time, developments went far to impress me with the opinion, that the disease, was not one of real hemorrhage, but of menstrual character. Upon instituting thorough inquiry into the history of the patient, I became satisfied of the nature of the case, and treated it accordingly. The attack soon passed off, and the patient recovered a pretty good state of health. My efforts were then directed to the correction of the uterine system and the establishment of healthy menstruation. In this, I have been partially, and only partially, successful. The catemenial secretion has become almost uniform in regard to the time of its recurrence, but is sometimes protracted and profuse, and not unfrequently attended with severe headache and distressing nausea. I have also observed a strong tendency on the part of the stomach, at each monthly period, to become nauseated, and, as I believe, to take on this remarkable action.

Before concluding this case, I ought to state, that it not unfrequently happens with this patient, that at her menstrual periods the breasts become more or less tumefied and painful, and at the same time similar appearances occur on other parts of the body, particularly on the chest.

The second case alluded to, is that of a negro woman, belonging to Mrs. ——, about thirty-five years of age, of apparently good constitution, and, with the exception about to be mentioned, general good health.

She began menstruating at the age of fifteen, and continued regular in this respect until about three years since. Eight years ago, when about twenty-seven years of age, she was attacked with violent pain in the foot, which was succeeded by an abscess, which was lanced, but did not heal. Ulceration succeeded, which continued to move upwards until the leg was involved and became the seat of its permanent location. About three years since, the catemenial discharge began manifestly to decline, and so continued until it ceased altogether, when she was seized with severe shooting pains, passing from the sacro lumbor, to the uterine region, and to the ovaries. At the approach of her next menstrual period, she noticed a slow oozing of blood from the ulcer on the leg, (I give her own account of the matter,) which continued about the usual time of that discharge and ceased. At subsequent *periods*, the same discharge sometimes occured, while at others, instead, small sacks of blood were formed contiguous to the ulcer, which were obliged to be opened and the blood discharged, before relief could be obtained.

In June last, the ulceration of the leg had become so extensive and threatening, as to require, in the judgement of Dr. ——, (whose patient she then was,) amputation.

Since the operation, the ulcer being removed, there has been no regular monthly periodic discharge of blood, but, at each monthly period, sacks, such as were above described, form around the stump of the amputated limb and require to be lanced for the relief of the patient. I have seen these sacks, and in fact opened them, and can entertain no doubt as to their true nature. So uniform are these singular occurrences in their periodic character, as to have induced this woman to keep a lancet for the purpose, and thus *surgically* to perfom the work of menstruation. It should be observed that she continues without any vaginal discharge, and that the determination of blood to the stump of the amputated limb, together with the formation of these sacks of blood, occur periodically, and observe *strictly* the menstrual periods, as to the time of their recurrence and duration.

